# Synthesis, In Silico Study, Antibacterial and Antifungal Activities of *N*-phenylbenzamides

**DOI:** 10.3390/ijms24032745

**Published:** 2023-02-01

**Authors:** Melanny Ika Sulistyowaty, Galih Satrio Putra, Tutuk Budiati, Anastasia Wheni Indrianingsih, Farida Anwari, Dini Kesuma, Katsuyoshi Matsunami, Takayasu Yamauchi

**Affiliations:** 1 Department of Pharmaceutical Sciences, Faculty of Pharmacy, Universitas Airlangga, Surabaya 60115, Indonesia; 2 Department of Chemistry, Faculty of Mathematics and Natural Sciences, Universitas Negeri Malang, Malang 65151, Indonesia; 3 Faculty of Pharmacy, Widya Mandala Catholic University, Surabaya 60265, Indonesia; 4 Research Center for Food Technology and Processing, National Research and Innovation Agency (BRIN), Yogyakarta 55861, Indonesia; 5 Medical Laboratory Science, University of Anwar Medika, Sidoarjo 61262, Indonesia; 6 Department of Pharmaceutical Chemistry, Faculty of Pharmacy, University of Surabaya, Surabaya 60293, Indonesia; 7 Graduate School of Biomedical and Health Sciences, Hiroshima University, 1-2-3 Kasumi, Minami-Ku, Hiroshima 734-8553, Japan; 8 Faculty of Pharmaceutical Science, Hoshi University, 2-4-41, Ebara, Shinagawa, Tokyo 142-8501, Japan

**Keywords:** synthesis, in silico, antibacterial, antifungal, *N*-phenylbenzamides derivatives, 3HAV, 2QZX

## Abstract

Antibiotic and antifungal resistance problems have been prevalent in recent decades. One of the efforts to solve the problems is to develop new medicines with more potent antibacterial and antifungal activity. *N*-phenylbenzamides have the potential to be developed as antibacterial and antifungal medicine. This study aimed to synthesize *N*-phenylbenzamides and evaluate their in silico and in vitro antibacterial and antifungal activities. The in silico studies conducted absorption, distribution, metabolism, excretion and toxicity (ADMET) predictions along with molecular docking studies. ADMET predictions used pkCSM software online, while the docking studies used MVD software (Molegro ^®^ Virtual Docker version 5.5) on Aminoglycosid-2 ″-phosphotransferase-IIa (APH2 ″-IIa) enzyme with protein data bank (PDB) ID code 3HAV as antibacterial and aspartic proteinases enzyme (Saps) with PDB ID code 2QZX as an antifungal. In vitro, antibacterial and antifungal tests were carried out using the zone of inhibition (ZOI) method. The five *N*-phenylbenzamides (**3a**–**e**) were successfully synthesized with a high yield. Based on in silico and in vitro studies, compounds **3a**–**e** have antibacterial and antifungal activities, where they can inhibit the growth of Gram-positive bacteria (*Staphylococcus aureus*), Gram-negative (*Escherichia coli*), and *Candida albicans*. Therefore, compounds **3a**–**e** can be developed as a topical antibacterial and antifungal agent.

## 1. Introduction

Infection is caused by pathogenic microorganisms such as bacteria, viruses, parasites or fungi. Infections are categorized into the top 10 deadliest diseases category. This disease can be transmitted directly or indirectly from one person to another [1]. This infectious disease also remains a significant problem in several countries in Africa, South America and south-east Asia [2,3] 

The prevalence of deadly diseases in the world and Indonesia is based on data from the WHO [1]. The WHO estimate that respiratory tract infections rank 4th, causing approximately 3.1 million individual deaths, or 5.5% of the total deaths in 2020. Digestive tract infections (diarrhea infections) were ranked 7th, which caused about 1.5 million individuals to die in 2020, or 2.7% of deaths worldwide, with about 2.2 million individuals dying. The statistical data of the National Basic Health Research issued by the Ministry of Health of Indonesia explained that there was a decrease in the incidence of gastrointestinal infections (diarrhea) from 9.0% in 2007 to 3.5% in 2017. However, there was an increase in the incidence of all-age pneumonia airway infections from 2.1% (2017) to 2.7% (2020) [4].

The discovery and use of antifungals and antibiotics for the first time in the 1900s caused the problem of antibiotic and antifungal resistance to occur in the 20th century [5]. Scientists and medical personnel must solve the problem of antibiotic and antifungal resistance by creating new compounds and modifying the structure of chemical compounds in previous antibiotics to reduce the incidence of antibiotic and antifungal resistance. One of the parent compounds that attracts the attention of researchers is *N*-phenylbenzamides. Previous studies have found that *N*-phenylbenzamides have antibacterial and antifungal activities [6,7,8]. In this study, we synthesized five *N*-phenylbenzamides and tested them in silico and in vitro for their antibacterial and antifungal activities.

In the in silico antibacterial assay, five *N*-phenylbenzamides were docked on Aminoglycosid-2 ″-phosphotransferase-IIa enzyme abbreviated as APH (2 ″) -IIa with the PDB ID code 3HAV, which is an enzyme produced by Gram-positive and Gram-negative bacteria to degrade the antibiotic potential of aminoglycosides such as gentamicin, kanamycin and streptomycin [9]. This enzyme is produced by bacteria resistant to aminoglycoside-class antibiotics. Streptomycin is a native ligand contained in this data bank protein, so streptomycin will also be used as a comparison standard in the in vitro antibacterial assay by diffusion method.

Streptomycin is an aminoglycoside antibiotic produced *by Streptomyces griseus* that has been found and used clinically from the 1940s to the present. Streptomycin has several advantages: it can be used in Gram-positive and Gram-negative aerobic bacteria such as *Yersinia pestis*, *Francisella tularensis*, *Calymmatobacterium granulomatis*, *H. influenza*, *K. pneumonia*, *E.coli*, *Proteus*, *A. aerogenes*, *K. pneumonia*, *Enterococcus faecalis*, *Streptococcus viridans*, *Enterococcus*. In addition, streptomycin also has activity in *mycobacterium tuberculosis*, becoming the second-line antituberculosis therapy for multi-drug-resistant tuberculosis [10,11].

In the in silico antibacterial assay, five *N*-phenylbenzamides were docked on aspartic proteinases (Saps), which are proteolytic enzymes produced by *Candida albicans* that act as a factor in the spread of infection/virulence of this species [12]. This enzyme functions as a proteolytic on the host tissue so that it is used as a place to attach *Candida albicans*. This enzyme does not only result in tissue damage in local candidiasis, but it can even reach the stage of systemic candidiasis [13,14,15]. In superficial candida infections, the barrier to the enzyme aspartic proteinases (Saps) is one of the enzyme targets to develop several antifungal drugs [12].

Pepstatin is a native ligand in aspartic proteinases in PDB ID code 2QZX. Pepstatin pentapeptide is isolated from *Streptomyces testaceous*. It is a potent inhibitor of aspartyl proteases with IC_50_ value = 27 nM. Pepstatin is not a drug used clinically for the treatment of candidiasis. However, in silico, in vitro or in vivo, pepstatin can inhibit the growth of superficial candida infections [13]. Micafungin has a more similar structure to pepstatin than imidazole antifungals (ketoconazole, chlortimazole, miconazole etc.) or benzylamine (terbinafine), so micafungin was selected for in silico and in vitro assay as an antifungal in the study [16].

In the in vitro antibacterial and antifungal activity assay, *Staphylococcus aureus* ATCC 6538 (Gram-positive bacteria), *Escherichia coli* ATCC 8739 (Gram-negative bacteria) and *Candida albicans* ATCC 10231 were selected. *Staphylococcus aureus* ATCC 6538 was used because it remains as the standard testing strain for disinfectants and antiseptics [17]. *Staphylococcus aureus* ATCC 29213 and ATCC 25923 were not chosen in the antibacterial assay testing because these two strains are used more as the standard laboratory testing control strain in the laboratory quality control [18]. *Escherichia coli* ATCC 8739 was used in this study because it is a pathogenic bacteria found in human feces that are often used for applications as a quality control strain in testing antimicrobial handwashing formulations, media testing, efficacy testing, and bioresistance testing. *Escherichia coli* ATCC 25922 was not used because that strain is commonly used as a quality control strain, particularly in antibody sensitivity assays [10]. *Escherichia coli* ATCC 35218 was also not chosen in the antibacterial activity test since it is used as the quality control organism for β-lactam–β-lactamase inhibitor agents [10]. *Candida albicans* ATCC 10231 was used because that strain is a normal flora in the human body; however, when the amount is excessive, it can cause pathogenic oral and vaginal candidiasis. *Candida albicans* ATCC 90028 was not used due to its functions mainly as the standard laboratory testing control strain in laboratory quality control [16].

## 2. Results and Discussion

### 2.1. Synthesis

**Figure 1:**
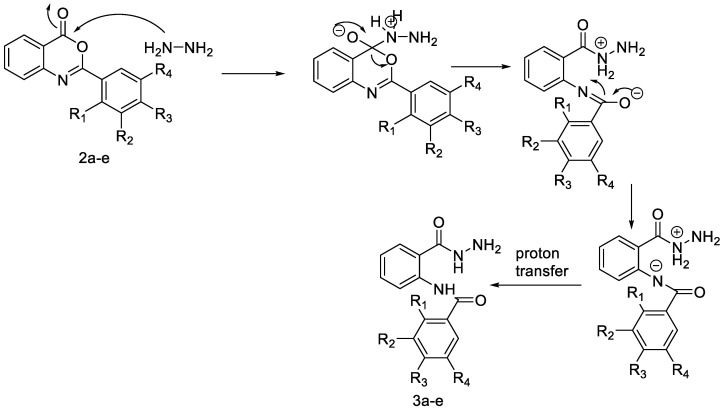
Attack of hydrazine hydrate of carbonyl against benzoxazine ring.

The process for the synthesis of *N*-phenylbenzamide derivative compounds was carried out in two reaction steps. The first step was where anthranilic acid was reacted with benzoyl chloride derivatives in a water-free solvent such as pyridine for 30 min to one hour to obtain benzoxazine derivatives. Our previously published articles explain the complete procedure [19,20]. The second step was to react the benzoxazine derivatives compound with hydrazine hydrate, refluxed for one hour or using a microwave irradiation of 600 W for one minute, and then *N*-phenylbenzamide derivatives were obtained. Our previously published articles explain the complete procedure [21,22]. The reaction mechanism between benzoxazine derivatives (**2a**–**e**) and hydrazine hydrate is the attack of nucleophile against hydrazine hydrate on the C carbonyl ring of the lactone, causing the opening of the lactone ring and forming amide groups so that *N*-phenylbenzamide derivatives were obtained. The recalibration mechanism is presented in Figure 1.

### 2.2. In Silico

#### 2.2.1. ADMET Predictions

The results of ADMET predictions of *N*-phenylbenzamide derivatives (**3a**–**e**) (Table 1) in the absorption aspect were not suitable through GIT because of the Caco2 permeability value <8 × 10^−6^ cm/s. Caco2 cell lines are human epithelial colorectal adenocarcinoma cells, which are cell monolayers often used as models of human intestinal mucosa in vitro to predict the oral absorption of drugs [23]. *N*-phenylbenzamides (**3a**–**e**) were not recommended orally because its bioavailability would be negligible. Only streptomycin antibiotic, an aminoglycoside antibiotic, seemed to have poor absorption in GIT. Therefore, there was only the formulations for injection and topical preparations. The results of skin permeability prediction of phenylbenzamide derivatives (**3b**–**e**) were outstanding for development in topical preparations because they had a log value of Kp < −2.5, which is included in the relatively good category of skin permeability for transdermal drug delivery. The phenylbenzamide derivative compounds (**3b**–**e**) were more suitable for topical preparations if they were active as an antibacterial in both positive and negative Grams and as an antifungal.

The predicted result of *N*-phenylbenzamide (**3a**–**e**) distribution (Table 1) indicates that it had a small volume of distribution with a value of <−0.15 log L/kg, meaning that the concentration of the drug in the plasma was more significant than in the tissue [23]. *N*-phenylbenzamide derivatives (**3a**–**e**) in the chemical structure of the drug studies were indeed included in the compounds that were hydrophiles because the amino and amide groups caused the lipophilic properties to be smaller. It was found that streptomycin and micafungin were very polar, so the drug was not distributed in fat tissue but circulated more in blood plasma [13]. Based on the study of the *N*-phenylbenzamide derivative compound (**3a**–**e**) distribution, it was predicted not to be able to penetrate BBB because it had a log BB value < −0.113. The blood–brain barrier is a protective monolayer of the brain, so chemical compounds do not quickly enter the brain [24]. Polar compounds with large molecular weights will certainly not be able to penetrate BBB [24].

The predicted results of the *N*-phenylbenzamide derivative compounds (**3a**–**e**) metabolism (Table 1) stipulated that it was not the drug inhibitor of CYP 2D6 and CYP 3A4. CYP 2D6 and CYP 3A4 are the two isoforms of cytochrome 450 in which almost 80% of the drug is metabolized by them [24]. Because *N*-phenylbenzamides (**3a**–**e**) are neither inducers nor inhibitors, they are safe because they do not interact much with other drugs. 

Based on the predicted results of the excretion using the pkCSM application (Table 1), the prediction of the rate of drug clearance (total clearance) in the body is a combination of hepatic clearance (liver metabolism and biliary clearance) and renal clearance [23,24,25]. The greater the total clearance, the faster the drug is secreted by the body. Compounds **3b**,**3d** and **3e** have a low total clearance rate compared to **3a** and **3b**. This might be because of the presence of halo substitution (Cl atoms) in benzene, so it is more difficult for it to be excreted by the body. Based on the toxicity prediction using the pkCSM application, compounds **3a**–**e** were not hepatotoxic and did not cause allergic reactions or hypersensitivity to the skin. Hence, they are safe for topical application.

The result of ADMET predictions summarized that compounds **3a**–**e** were not recommended to be made in the form of oral preparations because their absorption via the GIT is very low. However, it is acceptable for compounds **3a**–**e** to be developed as topical preparations since they have a good category of skin permeability for transdermal drug delivery and are not a skin irritant.

#### 2.2.2. Molecular Docking Studies

##### Antibacterial Docking Studies

**Figure 2:**
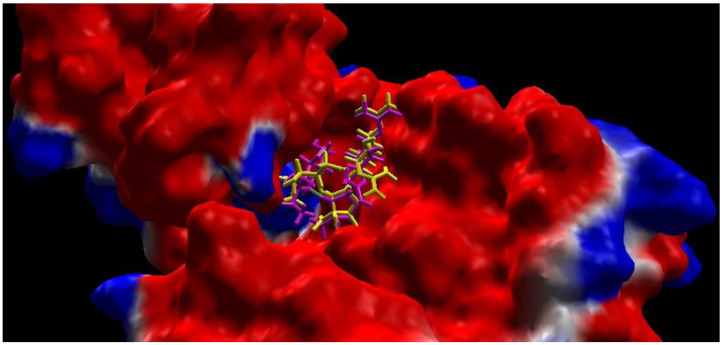
Comparison of the native ligand (streptomycin) (yellow) with the docking result simulation (purple) by Molegro Virtual Docker (MVD) Ver.5.5 with RMSD is 1.52 Å.

The grid box binding site was set X = −20.27 Å; Y = 8.05 Å; Z = −16.46 Å with a cavity surrounded by 43 amino acids, namely Lys 42; Arg 55; Glu 56; Lys 106; Glu 113; Lys 118; Lys 139; Lys 142; Asp 146; His 190; Asp 192; Phe 193; Ser 194; Asn 196; Asn 197; Asp 210; Asp 213; Asp 218; Asp 220; Asp 222; Leu 224; Cys 225; Asp 228; Ser 230; Asp 232; Asp 233; Lys 236; Arg 240; Lys 241; Lys 244; Glu 255; Arg 256; Lys 257; Glu 259; Asn 261; Asp 262; Tyr 264; Trp 265; Asp 268; Tyr 272; Arg 279; Lys 284; Glu 288. The result of redocking validation process with RMSD 1.52 Å is shown in Figure 2. Hence, these results indicate that the method is valid for the docking test of the tested compound since the RMSD obtained is less than 2 Å.

**Figure 3:**
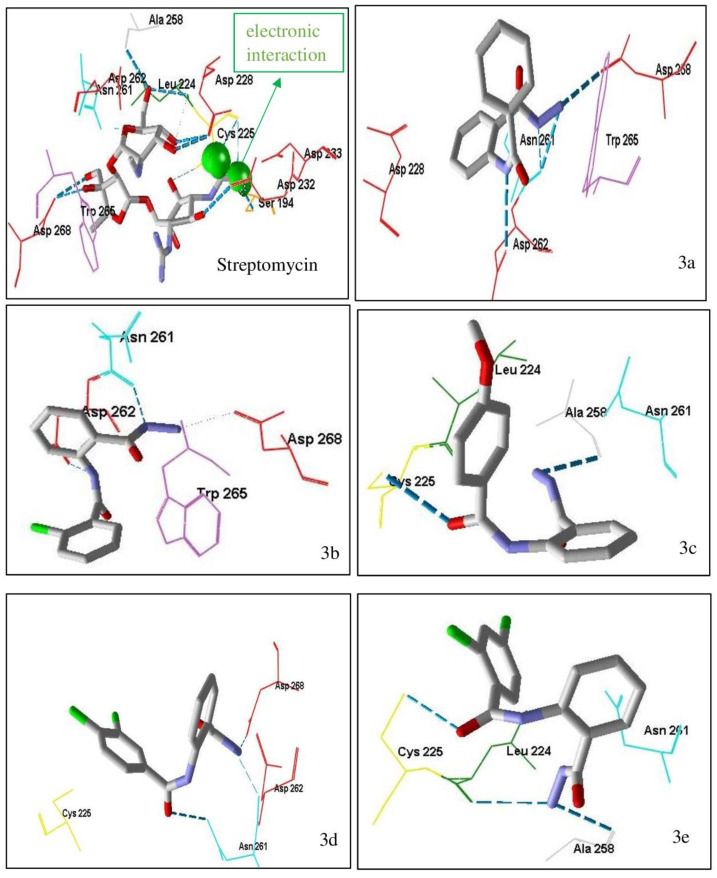
The molecular docking interaction between streptomycin and compounds **3a**–**e** into active site in Aminoglycosid-2 ″-phosphotransferase-IIa enzyme abbreviated APH (2 ″) -IIa methionyl-tRNA synthetase. Dashed line (blue color) is hydrogen bonding. The darker the blue color, the stronger hydrogen bonding and the closer the distance between the molecules. The details of color are as follows: yellow—cysteine (Cys); light blue—Asparagine (Asn); red—aspartic acid (Asp); dark green—leucine (Leu); dark pink—tryptophan (Trp); dark purple—tyrosine (Tyr); orange—serine (Ser); grey—alanine (Ala); white silver—glycine (Gly); dark blue—arginine (Arg); green—Isoleucine (Ile); peach—proline (Pro); light purple—histidine (His); light orange—threonine (Thr); sky blue—glutamine (Gln); maroon—glutamic acid (Glu); navy blue—lysine (Lys); light yellow—methionine (Met); magenta—phenylalanine (Phe); light green—valine (Val).

Based on the molecular docking studies of streptomycin in Aminoglycosid-2 ″-phosphotransferase-IIa enzyme abbreviated APH (2 ″)-IIa with PDB ID code 3HAV (Table 2 and Figure 3), streptomycin has hydrogen bond interaction, steric interaction and electronic interaction with moldock score value of −167.02 kcal/mol. These three interactions indicated that streptomycin had a shallow binding energy to the enzyme. Electronic bonding occurred in the aglycone group of streptomycin (amino group), which had a positive partial charge, to aspartate amino acid residues 192; 262 and 228, which had a negative partial charge.

There were several interactions of the hydrogen bond interaction of streptomycin with the enzyme APH (2 ″)-IIa by a bond distance of <3 Å, namely on the amino acid residue Asn 191 (C=O) with the H atom of the -OH group; two hydrogen bonds on Asn 261(NH) with the O atom of the C-O-C sugar group, and Asn 261 (C=O) with the H atom of the -OH group; Ala 258(C=O) with the 2 H atoms of the -OH group, and lastly on Asp 262 (C=O) with the amino group/ aglycone of streptomycin (NH), the rest of the hydrogen bonds being formed by a bond distance of >3 Å. The closer the distance between the hydrogen bonds, the lower the binding energy.

Some steric interactions occurred between streptomycin and the enzyme APH (2 ″)-IIa. Steric interaction is also known as hydrophobic interaction. The amino acid residues involved in steric interaction are Asn 191; Asn 221; Leu 224; Cys 225; Asp 228; Asp 262; Trp 265. Aspartate residues have an essential role in receptor drug bonds because there are three types of bonds: hydrogen bond, steric and electronic interaction.

The molecular docking result of compounds **3a**–**e** suggested that it had a greater moldock score than native ligand streptomycin, which was −99.91 until −92.08 kcal/mol. The binding energy of compounds **3a**–**e** was not as good as streptomycin on the enzyme APH (2 ″) -IIa. The benzamide and hydrazide groups, which were expected to act as amino aglycone groups in streptomycin, had electronic interactions with the target enzyme. In this case, aspartate residues were essential in drug-receptor interactions; compounds **3a**–**e** only interacted with hydrogen or steric bonds.

The presence of methoxy groups and halo, substituents in the phenylbenzamide derivatives provided energy-binding stability for the APH (2 ″) -IIa enzyme, although it was less significant.

##### Antifungal Docking Studies

**Figure 4:**
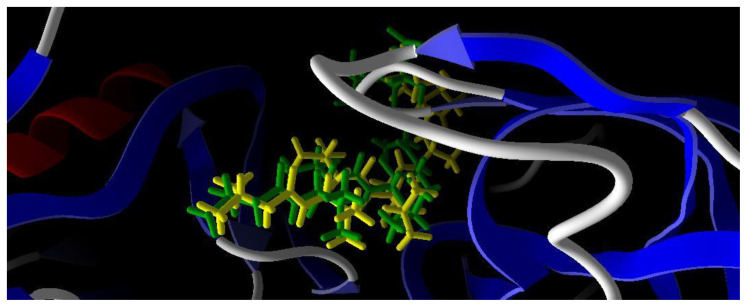
Comparison of its native ligand (pepstatin) (yellow) with the docking result simulation (green) by Molegro Virtual Docker (MVD) Ver.5.5 with RMSD is 1.89 Å.

The grid box binding site was set X = 8.51 Å; Y = 33.69 Å; Z = −25.99 Å with a cavity surrounded by 33 amino acids, namely Ile 12; Thr 13; Ile 30; Asp 32; Gly 34; Ser 35; Asp 37; Lys 50; Trp 51; Ile 82; Lys 83; Tyr 84; Gly 85; Asp 86; Ser 88; Lys 91; Arg 120; Ile 123; Gly 131; Glu 134; Lys 192; Lys 193; Asp 218; Gly 220; Thr 221; Thr 222; Ile 223; Tyr 225; Arg 297; Arg 299; Asp 303; Ile 305; Lys 323. The result of redocking validation process with RMSD 1.89 Å is shown in Figure 4. Hence, these results indicate that the method is valid for docking test of the tested compound since the RMSD obtained is less than 2 Å. 

Based on molecular docking studies (Table 3) of pepstatin in aspartic proteinases enzyme (Saps) with the PDB ID code 2QZX, pepstatin had hydrogen bond interaction and steric interaction with a binding energy value of −110.14 kcal/mol. Hydrogen bond interaction distance on amino acid residuals Lys 83, Gly 85, Gly 220 and Trp 221 is 2–3 Å. The closer the distance between hydrogen bonds, the lower the binding energy. Pepstatin also had steric interaction in several amino acid residues: Ile 12, Gly 34; Tyr 84; Asp 86, Asp 218 and Gly 220.

Micafungin, the standard antifungal drug in the in-silico test, had a much lower binding energy than the native ligand, which was −193.31 kcal/mol. The structure of micafungin caused the increase of hydrogen bonds and steric interaction in the enzyme aspartic proteinases. The hydrogen bond interaction in the residual amino acids Asp 32 and Ser 301 had a bond distance of <2 Å, while in other amino acid residues was 2–3 Å. In addition, the stability of micafungin bonds with the enzyme aspartic proteinases was also supported by steric interaction. The presence of several aromatic rings that bind to micafungin increases the amount of steric interaction in the enzyme.

**Figure 5:**
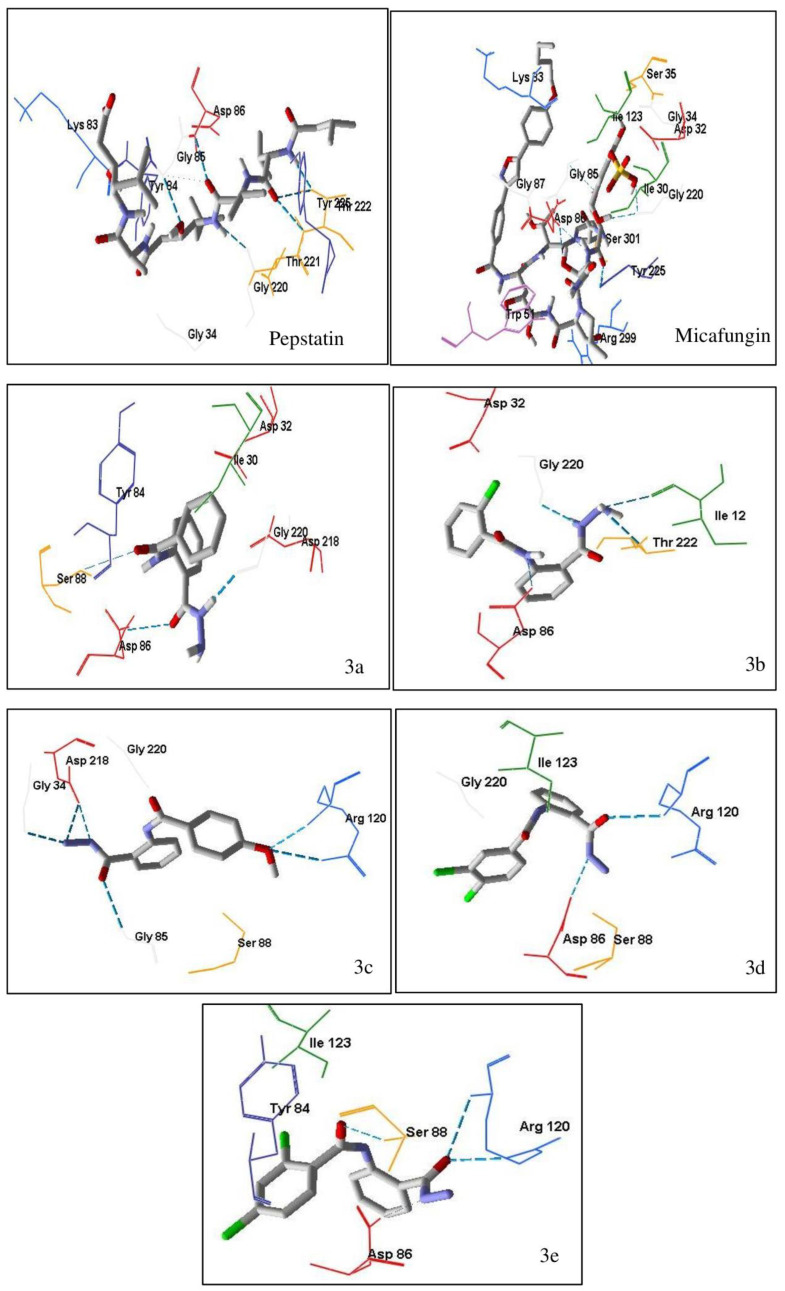
The molecular docking interaction between micafungin and compounds **3a**–**e** into active site in Aspartic proteinases (Saps). Dashed line (blue color) is hydrogen bonding. The darker the blue color, the stronger hydrogen bonding and the closer the distance between the molecules. The details of color are as follows: yellow—cysteine (Cys); light blue—Asparagine (Asn); red—aspartic acid (Asp); dark green—leucine (Leu); dark pink—tryptophan (Trp); dark purple—tyrosine (Tyr); orange—serine (Ser); grey—alanine (Ala); white silver—glycine (Gly); dark blue—arginine (Arg); green—Isoleucine (Ile); peach—proline (Pro); light purple—histidine (His); light orange—threonine (Thr); sky blue—glutamine (Gln); maroon—glutamic acid (Glu); navy blue—lysine (Lys); light yellow—methionine (Met); magenta—phenylalanine (Phe); light green—valine (Val).

Compounds **3a**–**e** had almost the same binding energy as a pepstatin-native ligand. The presence of halo substituents (**3b**, **3d**, **3e**) added low-energy binding to the target enzyme compared to the parent compound (**3a**). The presence of hydrazide and benzamide groups resulted in hydrogen bonds in several amino acids in aspartic proteinases. At the same time, the two aromatic rings acted as steric interactions in several amino acids (Figure 5).

#### 2.2.3. Antibacterial and Antifungal Activities

Based on the Clinical and Laboratory Standards Institute, streptomycin has an activity that is sensitive to *Staphylococcus aureus* with an inhibition zone of >15 mm, while for *Escherichia coli*, the inhibition zone is >14 mm. Streptomycin is categorized as resistant if it has an inhibition zone ≤12 mm. The results of the streptomycin antibiotic inhibition zone (10 µg) as a comparison standard have an inhibition zone of 29 mm both in *Staphylococcus aureus* and *Escherichia coli*, meaning that *Staphylococcus aureus* ATCC 6538 and *Escherichia coli* ATCC 8739 are still sensitive to the antibiotic. Streptomycin has inhibitory zone results with sensitive results in both because streptomycin has advantages; specifically, it can be used on both gram-positive and gram-negative aerobic bacteria [10].

Compounds **3a**–**e** (25 µg) have activity as an antibacterial in *Staphylococcus aureus* and *Escherichia coli* with an inhibition zone >14 mm are shown in Table 4, which is correlated sensitive to the bacteria but not as good as streptomycin, which remains as the comparison standard. In silico and in vitro results also prove that compounds **3a**–**e** are more inadequate than streptomycin in inhibiting the growth of *Staphylococcus aureus* and *Escherichia coli*. The presence of methoxy and halo substituents slightly increased their antibacterial activities compared to the parent compound (**3a**).

Based on the Clinical and Laboratory Standards Institute, micafungin has a sensitive activity to *Candida albicans* 14–23 mm [16]. The results of the micafungin antifungal inhibition zone (25 µg) as a comparison standard suggested that it had an inhibition zone of 23 mm, which means that *Candida albicans* used in the study were still sensitive to the antifungal.

**Figure 6:**
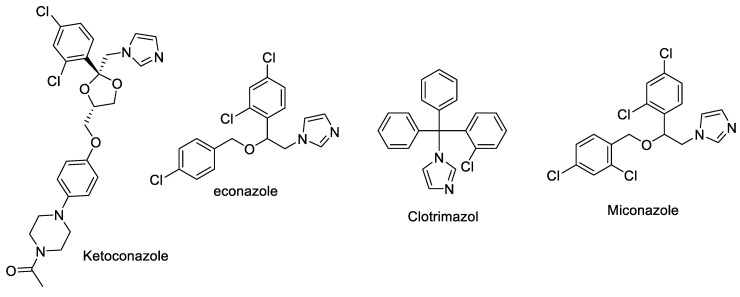
Chemical structure of azoles derivatives as antifungal.

Compounds **3a**–**e** (25 µg) have activity as an antifungal against *Candida albicans* with an inhibition zone >18 cm, which is categorized as sensitive but not as good as micafungin, which remains as the comparison standard. In silico and in vitro results also proved that compounds **3a**–**e** were inferior compared to micafungin in inhibiting the growth of *Candida albicans.* The presence of halo substituents slightly increased their antifungal activities compared to the parent compound (**3a**) or the methoxy substituent (**3c**). N atoms and atom Cl have an essential role in inhibiting the growth of *Candida albicans* because some of the imidazole group antifungal drugs (ex: ketoconazole, econazole, clotrimazole and miconazole) have been used by practitioners for oral and topical preparations for candidiasis cases (Figure 6).

**Figure 7:**
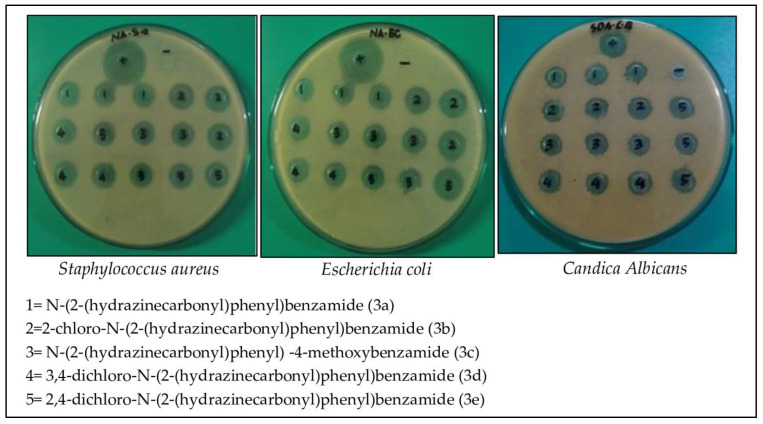
The result of antibacterial and antifungal activity by zone inhibition method.

Compounds **3a**–**e** have unique abilities to inhibit the growth of Gram-positive, negative and antifungal bacteria with a sensitive category even though the zone of inhibition is weaker than the respective comparative drug (Table 4 and Figure 7). The antibacterial activity against Gram-positive and Gram-negative is similar, and probably an indication of broad-spectrum antibiotics. However, the core of phenyl benzamide derivative compounds can be developed to be more potent as antibacterial and antifungal. Cases of superficial infection are currently experiencing many occurrences of cross-infection, namely infections caused by bacteria and fungi. Therefore, *N*-phenylbenzamide derivatives have the potential to be studied further.

## 3. Materials and Methods

### 3.1. Synthesis

**Figure 8:**
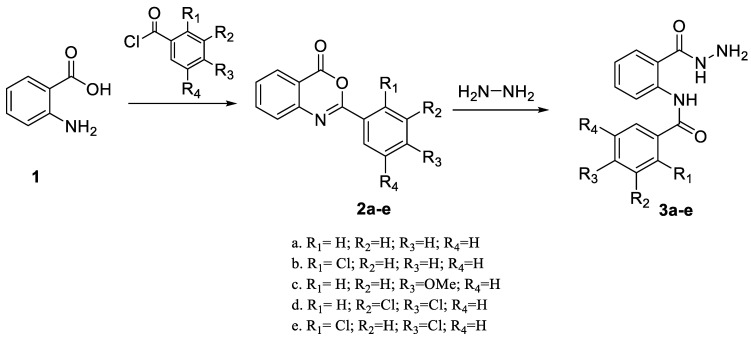
Synthesis of the title compounds.

Synthesis of *N*-phenylbenzamides was carried out in two reaction steps. The first reaction was to react the anthranilic acid with benzoyl chloride derivatives, producing benzoxazine derivatives. The second step was associating benzoxazine derivative compounds with hydrazine hydrate, as shown in Figure 8.

The first step was 10 mol of anthranilic acid dissolved in pyridine (O_2_-free solvent). It was then reacted with benzoyl chloride derivatives of 12.5 mol and stirred at a temperature of 0 °C for 1.5 h. The mixture formed was treated with a 10% NaHCO_3_ solution until no CO_2_ gas bubbles emerged. The solid formed was filtered off and washed with cold water. Then, the recrystallization process was carried out with 96% ethanol to obtain benzoxazine derivatives. The results of chemical physics and confirmation of chemical structure, including FT-IR, ^1^H-NMR, ^13^C-NMR, and MS, can be seen in our previous research [9,10].

The second step was to react 10 mol of benzoxazines with 10 mol of hydrazine hydrate, refluxed for one hour in an ethanol solvent or using a 600 W irradiation microwave for one minute in a DMSO solvent. Cold ethanol was added, and the solid was then obtained. Dissolved solids were filtered, and the recrystallization process was carried out with 96% ethanol [21,22].

The results of chemical physics and chemical structure elucidation of *N*-phenylbenzamide derivatives are presented below.

#### 3.1.1. N-(2-(hydrazinecarbonyl)phenyl)benzamide (**3a**)

It was obtained as a white powder with a yield of 99%, mp: 199–201 °C. FT-IR (KBr) cm^−1^: HN-C=O amide (1650, 3467); NH_2_-NH amine (701, 1258, 1607, 3318); C=C aromatic (1525, 2929). UV (λmax): 210, 228, 270 and 300 nm. ^1^H-NMR (600MHz, DMSO) δ 12.54 (s, 1H), 10.18 (s, 1H), 8.65 (d, *J* = 8.3 Hz, 1H), 7.96 (d, *J* = 7.6 Hz, 2H), 7.78 (d, *J* = 7.8 Hz, 1H), 7.64 (t, *J* = 7.1 Hz, 1H), 7.59 (t, *J* = 7.6 Hz, 2H), 7.56 (t, *J* = 7.6 Hz, 1H), 7.17 (t, *J* = 7.6 Hz, 1H). MS-ESI (m/z) = 278.0901 [M + Na]+ (calcd. for C_14_ H_13_ O_2_ N_3_ Na: 278.0900).

#### 3.1.2. 2-chloro-N-(2-(hydrazinecarbonyl)phenyl)benzamide (**3b**)

It was obtained as a white powder with a yield of 90%, mp: 191–192 °C. FT-IR (KBr) cm^−1^: HN-C=O amide (1673, 3214); NH2-NH amine (903, 1265, 1636, 3340); C=C aromatic (1049, 1509, 3116); aryl-Cl (1049). UV (λmax): 210, 214, 260, 296 nm. ^1^H-NMR (600MHz, DMSO) δ 11.89 (s, 1H), 10.10 (s, 1H), 8.56 (d, *J* = 8.2 Hz, 1H), 7.74 (d, *J* = 7.7 Hz, 1H), 7.67 (dd, *J* = 7.5, 1.3 Hz, 1H), 7.63–7.52 (m, 3H), 7.49 (t, *J* = 7.4 Hz, 1H), 7.20 (t, *J* = 7.6 Hz, 1H), 4.58 (s, 2H). ^13^C-NMR (151 MHz, DMSO) δ 167.04 (s), 164.32 (s), 138.49 (s), 136.27 (s), 131.99 (s), 131.75 (s), 130.20 (s), 129.87 (s), 128.91 (s), 127.68 (d, *J* = 10.5 Hz), 123.41 (s), 120.44 (s), 119.85 (s). MS-ESI (m/z) = 312.0513 [M + Na]+ (calcd. for C_14_ H_12_ O_2_ N_3_ Cl Na: 312.0510).

#### 3.1.3. N-(2-(hydrazinecarbonyl)phenyl)-4-methoxybenzamide (**3c**)

It was obtained as a white powder with a yield of 81%, mp: 194–195 °C. FT-IR (KBr) cm^−1^: HN-C=O amide (1651, 3311); NH2-NH amine (846, 1177, 1592, 3407); C=C aromatic (1037, 1507, 2059, 3017); -OCH_3_ methoxy (957, 1250). UV (λmax): 212, 278, 304 nm. ^1^H-NMR (600MHz, DMSO) δ 12.44 (s, 1H), 10.17 (s, 1H), 8.65 (d, *J* = 8.3 Hz, 1H), 7.92 (d, *J* = 8.7 Hz, 2H), 7.77 (d, *J* = 7.7 Hz, 1H), 7.53 (t, *J* = 7.8 Hz, 1H), 7.17–7.10 (m, 3H), 4.67 (s, 2H), 3.85 (s, 3H). ^13^C-NMR (151 MHz, DMSO) δ 168.06 (s), 164.36 (s), 162.72 (s), 139.95 (s), 132.53 (s), 129.34 (s), 128.10 (s), 127.14 (s), 123.04 (s), 120.58 (s), 119.35 (s), 114.65 (s), 55.97 (s), 40.41 (s), 40.27 (s), 40.13 (s), 40.01 (s), 39.99 (s), 39.78 (d, *J* = 21.0 Hz), 39.57 (s). MS-ESI (m/z)= 308.1007 [M + Na]+ (calcd. for C_15_ H_15_ O_3_ N_3_ Na: 308.1006).

#### 3.1.4. 3,4-dichloro-N-(2-(hydrazinecarbonyl)phenyl)benzamide (**3d**)

It was obtained as a white powder with a yield of 98%, mp: 214–215 °C. FT-IR (KBr) cm^−1^: HN-C=O amide (1666, 3384); NH_2_-NH amine (947, 1309, 1591, 3323); C=C aromatic (1529, 1743, 3063); aryl-Cl (1031). UV (λmax): 212, 238, 276 nm. ^1^H-NMR (600MHz, DMSO) δ 12.59 (s, 1H), 10.20 (s, 1H), 8.56 (d, *J* = 8.3 Hz, 1H), 8.11 (s, 1H), 7.89 (s, 2H), 7.79 (d, *J* = 7.7 Hz, 1H), 7.56 (t, *J* = 7.8 Hz, 1H), 7.20 (t, *J* = 7.6 Hz, 1H), 4.68 (s, 2H).^13^C-NMR (151 MHz, DMSO) δ 167.37 (s), 162.13 (s), 138.74 (s), 135.04 (s), 134.90 (s), 132.11 (s), 131.92 (s), 131.29 (s), 129.13 (s), 127.72 (s), 126.94 (s), 123.39 (s), 120.56 (s), 119.61 (s). HRMS-ESI (m/z) = 346.0121 [M + Na]+ (calcd. for C_14_ H_11_ O_2_ N_3_ Cl_2_ Na: 346.0121).

#### 3.1.5. 2,4-dichloro-N-(2-(hydrazinecarbonyl)phenyl)benzamide (**3e**)

It was obtained as a white powder with a yield of 91%, mp: 196–197 °C. FT-IR (KBr) cm^−1^: HN-C=O amide (1664, 3263); NH_2_-NH amine (943, 1120, 1596, 3447); C=C aromatic (1049, 1524, 1935, 3053); aryl-Cl (1103). UV (λmax): 210, 262, 294 nm. ^1^H-NMR (600MHz, DMSO) δ 11.90 (s, 1H), 10.10 (s, 1H), 8.49 (d, *J* = 8.1 Hz, 1H), 7.79 (d, *J* = 1.8 Hz, 1H), 7.73 (d, *J* = 7.7 Hz, 1H), 7.71 (d, *J* = 8.2 Hz, 1H), 7.59 (dd, *J* = 8.2, 2.0 Hz, 1H), 7.56 (t, *J* = 7.8 Hz, 1H), 7.21 (t, *J* = 7.6 Hz, 1H), 4.60 (s, 2H). ^13^C-NMR (151 MHz, DMSO) δ 166.94 (s), 163.38 (s), 135.55 (s), 135.08 (s), 131.95 (s), 131.15 (s), 130.34 (s), 129.74 (s), 127.87 (s), 127.71 (s), 123.59 (s), 120.61 (s). MS-ESI (m/z) = 346.0126 [M + Na]+ (calcd. for C_14_ H_11_ O_2_ N_3_ Cl_2_ Na: 346.0121).

### 3.2. Synthesis

#### 3.2.1. ADMET Prediction Studies

Compounds of *N*-phenylbenzamide derivatives (**3a**–**e**), streptomycin, and micafungin were drawn using a Marvin sketch and stored as a smile. The data were included in the online pcKSM software to obtain ADMET prediction data [23].

#### 3.2.2. Molecular Docking Studies

The in silico study was conducted using MVD software (Molegro ^®^ Virtual Docker version 5.5). The compound to be stocked was stored in 3D geometry with the lowest energy set of MMFF94 mode [26]. In the in silico study as an antibacterial, it was stocked against the enzyme Aminoglycosid-2’’-phosphotransferase-IIa (APH2 ″-IIa) with the PDB ID code 3HAV. Gram-positive and negative bacteria produced this enzyme to deplete the aminoglycoside antibiotic class [9]. In the in silico study as an antifungal, it was stocked against the aspartic proteinases enzyme (Saps) with the PDB ID code 2QZX. Aspartic proteinases are proteolytic enzymes produced by *Candida albicans* that play a role in proteolytic host tissues so that they are used as a place to attach *Candida albicans* [12].

The in silico test is validated as an antibacterial by redocking the native ligand (streptomycin) on the active side of the enzyme APH2 ″-IIa. Meanwhile, to test the antifungal, we did the redocking of the native ligand (pepstatin) on the active side of the enzyme aspartic proteinases. The acceptance criteria were set with the value of RMSD ≤ 2.0 Å.

The evaluation was carried out using the moldock score. The smaller the moldock score, the more stable binding between ligand and receptor [27]. The moldock score is the total energy from external and internal ligand interaction. External ligand interaction is a sum of the energy consistency of protein–ligand interaction and cofactor–ligand interaction. Internal energy interaction is a sum of energy dependent on the chemical structures of the ligand, such as torsional strain, torsional strain sp2-sp2 steric and electrostatic.

### 3.3. In Vitro Assay

#### 3.3.1. Antibacterial Activity Studies

An in vitro study of the antibacterial activity was performed on the diffusion disk. Gram-positive bacteria *Staphylococcus aureus* ATCC 6538 and Gram-negative bacteria *Escherichia coli* ATCC 8739 were used in the study. Mueller-Hinton recommended that a media made with an aseptic technique and streptomycin (10 µg) was used as a positive control following CLSI guidelines. The standard aminoglycosides class can be used according to CLSI is 10–30 µg, depending on the type of antibiotic used (ex: gentamicin 10 µg, amikacin 30 µg, kanamycin 30 µg, netilmicin 30 µg, tobramycin 10 µg). Compounds **3a**–**e** were dissolved at a ratio of ethanol and DMSO (1:9) with a level of 25 µg, respectively. The antibacterial test was performed in three replications and incubated in an incubator at a temperature of 32.5 °C for 24 h. After the incubation period, the sample was removed from the incubator and the size of the formed zone was measured by calculating the shelf life [28].

#### 3.3.2. Antifungal Activity Studies

An in vitro antifungal activity study was performed on the diffusion disk. *Candida albicans* (ATCC 10231) was used for the activity test. Sabouraud media with aseptic and micafungin techniques (25 µg) was used as a positive control following CLSI guidelines. Compounds **3a**–**e** were dissolved at a comparison of ethanol and DMSO (1:9) with a level of 25 µg, respectively. The antibacterial activity test was performed in three replications and incubated at 20–25 °C for 48 h. After the incubation period, the sample was removed from the incubator, and the size of the formed zone was measured by calculating the shelf life [28].

## 4. Conclusions

Compounds **3a**–**e**, based on the results of in silico and in vitro studies, were not as good as the standard drugs; however, their activities were still categorized as sensitive on the Gram-positive bacteria (*Staphylococcus aureus*), Gram-negative (*Escherichia coli*) and *Candida albicans.* Therefore, compounds **3a**–**e** are interesting to be developed as topical antibacterial and antifungal agent preparations, because there are only a few chemical compounds that have both antibacterial and antifungal activity.

## Figures and Tables

**Table 1:** The result of ADMET Predictions.

Compounds	Absorption		Distribution		Metabolism		Excretion	Toxicity		
	Caco2 Perm. log Papp in 10^−6^ cm/s	Skin Perm. log Kp	VDss (log L/kg)	BBB Perm. (BB log)	CYP2D6 Inhibitor	CYP3A4 Inhibitor	Total Clearance log mL/min/kg	Oral Rat Acute Toxicity mol/kg (LD50)	Hepato toxicity	Skin Sensiti-zation
**3a**	0.699	0.699	−0.545	0.93	No.	No.	0.185	2.242	No.	No.
**3b**	0.782	−2.743	0.571	−1.094	No.	No.	−0.019	2.243	No.	No.
**3c**	0.362	−2.748	−0.417	−1.146	No.	No.	0.161	2.195	No.	No.
**3d**	0.922	−2.744	0.49	−1.284	No.	No.	0.064	2.255	No.	No.
**3e**	0.876	−2.744	−0.583	−1.279	No.	No.	0.058	2.247	No.	No.
Streptomycin	−0.585	−0.585	−1.031	−2.872	No.	No.	–0.022	2.444	No.	No.
Micafungin	0.906	−2.735	−0.528	−4.305	No.	No.	1.18	2.482	Yes	No.

**Table 2:** The Molecular docking result of Streptomycin and compounds **3a**–**e** into active site Aminoglycosid-2’’-phosphotransferase-IIa enzyme.

Compounds	Moldock Score (kcal/mol)	Hydrogen Bond Interaction	Steric Interaction	Electronic Interaction
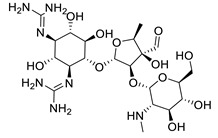 Native ligand (streptomycin)	−167.02	Try 272 Asn 191 Ala 258 Ser 230 Asp 228 Cys 255 Asn 261 Asp 262	Trp 265 Asn 191 Asn 221 Leu 224 Asp 262 Asp 228 Cys 225	Asp 192 Asp 262 Asp 228
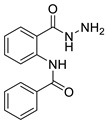 **3a**	−92.08	Asn 261 Asp 268 Asp 262	Asp 228 Trp 265	-
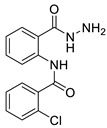 **3b**	−94.59	Asp 262 Asn 261	Asn 261 Asp 268 Trp 265	-
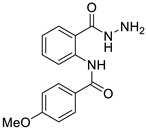 **3c**	−98.19	Ala 258	Asn 261 Leu 224 Cts 225	
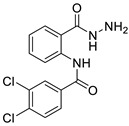 **3d**	−99.91	Asn 261 Asp 268	Asp 262 Cys 225	-
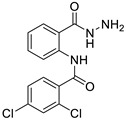 **3e**	−98.63	Cys 255 Leu 224 Ala 258	Cys 255 Asn 261	-

**Table 3:** The Molecular docking result of micafungin and compounds **3a**–**e** into aspartic proteinases.

Compounds	Moldock Score (kcal/mol)	Hydrogen Bond Interaction	Steric Interaction	Electronic Interaction
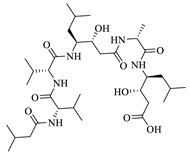 Native ligand (pepstatin)	−110.14	Lys 83 Gly 85 Gly 220 Trp 221	Gly 34 Tyr 84 Gly 220 Asp 218 Asp 86 Ile 12	-
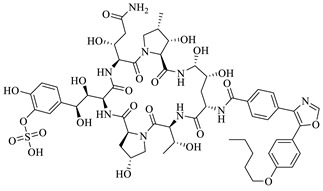 Micafungin	−193.31	Ser 301 Tyr 225 Asp 86 Gly 220 Asp 32 Ser 301	Gly 85 Arg 299 Gly 87 Lys 83 Asp 32 Gly 34 Ser 35 Ile 30 Ile 123 Ser 301 Asp 86 Trp 51	-
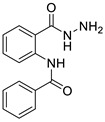 **3a**	−102.5	Asp 86 Gly 220 Ser 88	Asp 218 Tyr 84 Asp 32 Ile 30	-
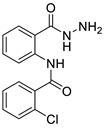 **3b**	−106.35	Asp 86 Gly 220 The 222 Ile 12	Asp 32 Asp 86	-
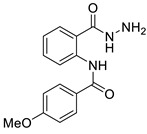 **3c**	−105.88	Gly 85 Arg 120 Asp 218	Ser 88 Gly 220 Gy 34	
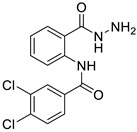 **3d**	−109.14	Ser 88 Asp 86 Arg 120	Ile 123 Gly 220	-
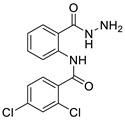 **3e**	−106.46	Ser 88 Asp 86 Arg 120	Ile 123 Tyr 84 Arg 120	-

**Table 4:** The result of antibacterial and antifungal activity.

Compounds	Gram-Positive Antibacterial (mm)	Gram-Negative Antibacterial (mm)	Antifungal (mm)
	*Staphylococcus aureus* (ATCC 6538)	*Escherichia coli* (ATCC 8739)	*Candica albicans* (ATCC 10231)
**3a** (25 µg)	16.12 ± 0.01	16.14 ± 0.02	17.18 ± 0.01
**3b** (25 µg)	17.12 ± 0.01	16.15 ± 0.01	18.19 ± 0.01
**3c** (25 µg)	16.13 ± 0.01	16.12 ± 0.01	17.17 ± 0.01
**3d** (25 µg)	16.14 ± 0.01	16.13 ± 0.01	18.17 ± 0.01
**3e** (25 µg)	16.13 ± 0.01	15.19 ± 0.01	18.18 ± 0.02
Streptomycin (10 µg)	29.14	29.17	-
Micafungin (25 µg)	-	-	23.18

## Data Availability

The data written in this study are accessible on request from the corresponding authors.
